# Prediagnostic Plasma Nutrimetabolomics and Prostate Cancer Risk: A Nested Case–Control Analysis Within the EPIC Study

**DOI:** 10.3390/cancers16234116

**Published:** 2024-12-08

**Authors:** Enrique Almanza-Aguilera, Miriam Martínez-Huélamo, Yamilé López-Hernández, Daniel Guiñón-Fort, Anna Guadall, Meryl Cruz, Aurora Perez-Cornago, Agnetha L. Rostgaard-Hansen, Anne Tjønneland, Christina C. Dahm, Verena Katzke, Matthias B. Schulze, Giovanna Masala, Claudia Agnoli, Rosario Tumino, Fulvio Ricceri, Cristina Lasheras, Marta Crous-Bou, Maria-Jose Sánchez, Amaia Aizpurua-Atxega, Marcela Guevara, Kostas K. Tsilidis, Anastasia Chrysovalantou Chatziioannou, Elisabete Weiderpass, Ruth C. Travis, David S. Wishart, Cristina Andrés-Lacueva, Raul Zamora-Ros

**Affiliations:** 1 Unit of Nutrition and Cancer, Cancer Epidemiology Research Program, Catalan Institute of Oncology (ICO), Bellvitge Biomedical Research Institute (IDIBELL), 08908 Barcelona, Spain; ealmanza@idibell.cat (E.A.-A.); dguinon@idibell.cat (D.G.-F.); marta.crous@iconcologia.net (M.C.-B.); 2 Centro de Investigación Biomédica en Red de Fragilidad y Envejecimiento Saludable (CIBERFES), Instituto de Salud Carlos III, 28029 Madrid, Spain; mmartinezh@ub.edu (M.M.-H.); annaguadall@ub.edu (A.G.); mcruzb@ub.edu (M.C.); candres@ub.edu (C.A.-L.); 3 Biomarkers and Nutrimetabolomics Laboratory, Department of Nutrition, Food Sciences and Gastronomy, Nutrition and Food Safety Research Institute (INSA), Food Innovation Network (XIA), Faculty of Pharmacy and Food Sciences, University of Barcelona, 08028 Barcelona, Spain; 4 The Metabolomics Innovation Centre, University of Alberta, Edmonton, AB T6G 1C9, Canada; yamile@ualberta.ca (Y.L.-H.); dwishart@ualberta.ca (D.S.W.); 5 CONAHCyT-Metabolomics and Proteomics Laboratory, Academic Unit of Biological Sciences, Autonomous University of Zacatecas, Zacatecas 98000, Mexico; 6 Cancer Epidemiology Unit, Nuffield Department of Population Health, University of Oxford, Oxford OX3 7LF, UKruth.travis@ndph.ox.ac.uk (R.C.T.); 7 Danish Cancer Society Research Center, Diet, Cancer and Health, 2100 Copenhagen, Denmark; agnrha@cancer.dk (A.L.R.-H.); annet@cancer.dk (A.T.); 8 Department of Public Health, University of Copenhagen, 1353 Copenhagen, Denmark; 9 Department of Public Health, Aarhus University, 8000 Aarhus, Denmark; ccd@ph.au.dk; 10 Department of Cancer Epidemiology, German Cancer Research Center (DKFZ), 69120 Heidelberg, Germany; v.katzke@dkfz-heidelberg.de; 11 Department of Molecular Epidemiology, German Institute of Human Nutrition Potsdam Rehbruecke, 14558 Nuthetal, Germany; mschulze@dife.de; 12 Institute of Nutritional Science, University of Potsdam, 14558 Nuthetal, Germany; 13 Clinical Epidemiology Unit, Institute for Cancer Research, Prevention and Clinical Network (ISPRO), 50139 Florence, Italy; g.masala@ispro.toscana.it; 14 Department of Research Epidemiology and Prevention Unit, Fondazione IRCCS Istituto Nazionale Dei Tumori, 20133 Milan, Italy; claudia.agnoli@istitutotumori.mi.it; 15 Hyblean Association for Epidemiological Research, Associazione Iblea per la Ricerca Epidemiologica (AIRE-ONLUS), 97100 Ragusa, Italy; rosario.tumino@asp.rg.it; 16 Centre for Biostatistics, Epidemiology, and Public Health (C-BEPH, Department of Clinical and Biological Sciences, University of Turin, Orbassano, 10043 Turin, Italy; fulvio.ricceri@unito.it; 17 Department of Functional Biology, Oviedo University, 33003 Oviedo, Spain; lasheras@uniovi.es; 18 Escuela Andaluza de Salud Pública (EASP), 18011 Granada, Spain; mariajose.sanchez.easp@juntadeandalucia.es; 19 Instituto de Investigación Biosanitaria ibs.GRANADA, 18012 Granada, Spain; 20 Centro de Investigación Biomédica en Red de Epidemiología y Salud Pública (CIBERESP), 28029 Madrid, Spain; mp.guevara.eslava@navarra.es; 21 Sub Directorate for Public Health and Addictions of Gipuzkoa, Ministry of Health of the Basque Government, 20013 San Sebastian, Spain; a-aizpuruaatxega@euskadi.eus; 22 Epidemiology of Chronic and Communicable Diseases Group, Biogipuzkoa (BioDonostia) Health Research Institute, 20014 San Sebastián, Spain; 23 Instituto de Salud Pública y Laboral de Navarra, 31003 Pamplona, Spain; 24 Navarra Institute for Health Research (IdiSNA), 31008 Pamplona, Spain; 25 Department of Epidemiology and Biostatistics, School of Public Health, Imperial College London, St Mary’s Campus, London SW7 2AZ, UK; k.tsilidis@imperial.ac.uk; 26 International Agency for Research on Cancer (IARC/WHO), 69366 Lyon, France; chatziioannouc@iarc.who.int (A.C.C.); weiderpasse@iarc.who.int (E.W.); 27 Department of Biological Sciences, University of Alberta, Edmonton, AB T6G 1C9, Canada

**Keywords:** EPIC, nutrimetabolomics, nested case–control, prostate cancer

## Abstract

**Background and Objective**: Nutrimetabolomics may reveal novel insights into early metabolic alterations and the role of dietary exposures on prostate cancer (PCa) risk. We aimed to prospectively investigate the associations between plasma metabolite concentrations and PCa risk, including clinically relevant tumor subtypes. **Methods**: We used a targeted and large-scale metabolomics approach to analyze plasma samples of 851 matched PCa case–control pairs from the European Prospective Investigation into Cancer and Nutrition (EPIC) cohort. Associations between metabolite concentrations and PCa risk were estimated by multivariate conditional logistic regression analysis. False discovery rate (FDR) was used to control for multiple testing correction. **Results**: Thirty-one metabolites (predominately derivatives of food intake and microbial metabolism) were associated with overall PCa risk and its clinical subtypes (*p* < 0.05), but none of the associations exceeded the FDR threshold. The strongest positive and negative associations were for dimethylglycine (OR = 2.13; 95% CI 1.16–3.91) with advanced PCa risk (n = 157) and indole-3-lactic acid (OR = 0.28; 95% CI 0.09–0.87) with fatal PCa risk (n = 57), respectively; however, these associations did not survive correction for multiple testing. **Conclusions**: The results from the current nutrimetabolomics study suggest that apart from early metabolic deregulations, some biomarkers of food intake might be related to PCa risk, especially advanced and fatal PCa. Further independent and larger studies are needed to validate our results.

## Simple Summary

Previous studies using a metabolomics approach in relation to prostate cancer (PCa) risk assessment have focused on endogenous metabolism. As far as we know, the associations between circulating metabolites derived from exogenous sources, such as diet or lifestyle, and PCa risk have not been widely studied. In our work, we examined the associations between endogenous and exogenous plasma metabolites and PCa risk in a case–control study conducted in the frame of the EPIC study. The main findings of our study include novel negative and positive associations of food metabolites, such as cyclamate and biomarkers of plant-based foods, respectively, with PCa cancer risk. Furthermore, a few microbial metabolites were associated with PCa risk, suggesting the potential role of microbiota in prostate cancer development. Our results reinforce the use of nutritional metabolomics to deepen the understanding of the complex role of diet and its potential mechanisms of action in prostate carcinogenesis.

## 1. Introduction

Prostate cancer (PCa) is the second-most frequent malignancy in men worldwide. According to GLOBOCAN, 1,414,000 new cases of PCa were recorded and 375,304 PCa deaths occurred in 2020 [1]. PCa is a disease that displays notable diversity in clinical, morphological, and molecular aspects [2]. Age, ethnicity, and family history are well-known non-modifiable risk factors that play a key role in the development of PCa [3], while modifiable risk factors such as physical activity and metabolic syndrome are suggestive intermediate risk factors, especially between coronary heart diseases and PCa [4]. However, the role of diet, obesity, and smoking are still not completely understood [5]. In this context, the systematic quantification and characterization of low-molecular-weight compounds in body fluids through the use of metabolomics may help to not only identify novel biomarkers of PCa risk but to also dissect the role of internal (e.g., genome) and external risk factors (diet, lifestyle, environment, gut microbiota) in PCa pathogenesis and progression.

Over the last decade, metabolomics has been extensively employed in various endeavors aimed at identifying novel biomarkers of PCa risk [6]. Epidemiological studies using metabolomics in relation to PCa risk have predominately used a targeted approach and measured endogenous metabolites [7]. Specially, the associations between circulating concentrations of metabolites related to energy, amino acid and lipid metabolism, and PCa risk have previously been reported in four nested case–control studies within the European Prospective Investigation into Cancer and Nutrition (EPIC) cohort [8,9,10,11]. Briefly, the findings included either positive or inverse associations for individual metabolites and metabolite patterns comprised of acylcarnitines, amino acids, biogenic amines, fatty acids, glycerophospholipids, hexoses, and sphingolipids with PCa risk, including overall, advanced, and fatal subtypes. As far as we know, the associations between circulating metabolites derived from exogenous sources such as diet or lifestyle and PCa risk have not been widely studied, and in the case of the EPIC cohort, have not been investigated.

In the present study, we aimed to investigate the prospective association between endogenous and exogenous plasma metabolite concentrations and the risk of overall PCa and PCa clinical subtypes in a nested case–control study within the EPIC cohort. In order to find possible nutritional biomarkers related to PCa risk, a large-scale targeted nutrimetabolomics approach was carried out for the detection and quantification of diet-related metabolites. Furthermore, to add evidence on metabolic alterations preceding the development and progression of PCa, we measured a series of endogenous metabolites, including some not previously reported in previous EPIC studies.

## 2. Materials and Methods

### 2.1. Study Population

EPIC is an on-going multi-center and prospective study designed to investigate the relationships between diet, lifestyle, environmental, genetic factors, and cancer risk. From 1992 to 1998, the EPIC study enrolled over 153,000 men aged 35–70 y, mostly from the general population of 19 centers in 8 western European countries (Denmark, Germany, Greece, Italy, The Netherlands, Spain, Sweden, and the United Kingdom). Details of the EPIC study have been previously described [12,13]. Non-fasting blood samples from 139,600 men were collected at baseline and immediately stored in liquid nitrogen tanks (−196 °C) at the central biobank of the International Agency of Research on Cancer (IARC; Lyon, France) and associated centers in Germany, Greece, Italy, The Netherlands, Spain, and United Kingdom. Since only women were recruited in France, Norway, Naples (Italy), and Utrecht (The Netherlands), these centers were not included in the current study, and male participants’ data from Germany, Italy, Spain, The Netherlands, and the United Kingdom were included in the current analysis. Participants’ blood samples and data from Denmark, Greece, and Sweden were not available for the current study. In addition to the data and blood samples’ availability, participants were eligible if the date of blood collection was known, and if they had no prevalent cancer other than non-melanoma skin cancer at the time of blood collection. All EPIC study procedures were reviewed and approved by the ethical review board of the IARC and the local ethics committees in the participating countries. All participants gave written informed consent prior to the study.

### 2.2. Follow-Up and Case–Control Selection

Information on PCa incidence and tumor subtypes was gathered by linking records to cancer registries at both the regional and national level, with the exception of Germany where a combination of methods was used. The data include health insurance records, cancer and pathology monitoring, and active follow-up. Self-reported incident cancers were confirmed through medical records and the vital status of each participant was collected from regional or national mortality registries. The cases included male patients diagnosed with first primary and incident PCa (defined as code C61 in the 10th revision of the International Statistical Classification of Diseases and Related Health Problems [ICD-10]) after blood collection and before the end of follow-up in August 2014. Incident PCa cases were matched to male controls by age (±6 months), study center, length of follow-up period, time of day (±1.0 h), and fasting status (<3.0, 3.0–6.0, >6.0 h) at blood collection in a nested case–control study design. Controls were randomly selected from male cohort participants who were alive and without cancer (excluding non-melanoma skin cancer) at the time the cases were diagnosed. A sampling method was used to determine incidence density, where a control could become a case later or be a control for more than one case.

PCa subtypes were classified according to the tumor–node–metastasis (TNM) system and histological grade in the following categories: localized (tumors confined within the prostate with no metastasis upon diagnosis; ≤T_2_ and N_0/x_ and M_0_, or coded as localized, n  =  303), advanced (tumors that had spread beyond the prostate at diagnosis; T_3–4_ and/or N_1–3_ and/or M_1_, or coded as advanced, n  =  157), low–intermediate (Gleason score < 8, or grade coded as well, moderately, or poorly differentiated, n = 589), and high grade (Gleason score ≥ 8, or grade coded as undifferentiated, n = 116). Fatal cases were defined as PCa listed as the underlying cause of death on the death certificate during the follow-up (n  =  55).

### 2.3. Dietary, Lifestyle, and Anthropometrics Assessment

Habitual dietary data, including alcohol intake during the year before recruitment, were collected using validated center-specific self-reported dietary questionnaires [12,13]. Also, at the time of enrollment, participants provided detailed information on their lifestyle, including smoking status and physical activity, sociodemographic factors, and medical history through standardized questionnaires. Anthropometric measurements were taken by trained professional study personnel in all EPIC centers, except for the Oxford center (UK), where participants reported their own anthropometrics measurements, which were later on validated [12,14].

### 2.4. Targeted Metabolomics Analysis and Quality Control Assessment

Plasma samples were leftovers from a previous EPIC study. Therefore, samples experienced one freeze–thaw cycle before analysis and had been stored at −80 °C. Plasma samples were analyzed using the multi-analyte targeted metabolomics platform developed at the Nutrimetabolomics laboratory of the University of Barcelona [15], which enables the simultaneous detection and quantification of more than 1000 compounds from endogenous and exogenous origin. For the current study, we analyzed a subset of 591 compounds, including metabolites from the endogenous (n = 146, 25%) and exogenous metabolome (n = 445, 75% [e.g., diet, smoking, alcohol consumption]). The rationale for including these compounds in that proportion was based on the following two observations: (i) endogenous metabolites were selected because they are involved in the main metabolic pathways (lipid, amino acid, glucose, and tricarboxylic acids metabolism) but at the same time differ from those analyzed in previous EPIC sub-studies; and (ii) exogenous metabolites were selected to test new associations as well as others previously observed in observational and experimental studies. Diet-derived compounds comprised (poly)phenols, artificial sweeteners, xanthines, alkaloids, and their phase I/II metabolites, as well as gut microbiota-transformed derivatives. Of note, we included the analysis of four glucosinolates (glucoberteroin, glucobrassicanapin, glucolepidiin, and glucoraphanin) and four isothiocyanates (allyl isothiocyanate, butyl isothiocyanate, phenethyl isothiocyanate, and benzyl isothiocyanate), whose presence (either through their dietary intake estimation or isolated forms in human PCa cell lines) has been suggested to reduce PCa risk [16,17].

In short, plasma samples were thawed at 4 °C, then proteins were precipitated using Sirocco Plates (Waters, Milford, MA, USA) before transferring the supernatants to a 96-well injection plate along with 14 isotopically labeled external standards. Ultra-high-performance liquid chromatography (1290 Infinity UHPLC System; Agilent, Santa Clara, CA, USA) coupled to tandem mass spectrometry (MS/MS) QTRAP 6500 (Sciex, Framingham, MA, USA) was used to conduct analyses, using the operating conditions described elsewhere [15]. Calibration curves were created for all analyzed metabolites at 12 different concentrations between 0.10 and 4000 μg/L. Analyst 1.6.2 and SCIEX OS-Q 2.1 software (ciex, Framingham, MA, USA) were used for data acquisition and processing, respectively.

Metabolite data (expressed as ng/mL) were preprocessed using an in-house pipeline that uses the POMA [18] and metaboprep [19] R/Bioconductor packages. Data preprocessing included the removal of any metabolite with a coefficient of variation ≥30% in internal quality controls, and of endogenous and exogenous metabolites with >20% and >90% of missing values, respectively. The remaining missing values were imputed to zero. These filtering steps resulted in the removal of 445 metabolites, thus leaving a total of 146 metabolites (45 endogenous and 101 exogenous) for further analysis (Supplementary Figure S1). Filtered data were log2-transformed after the addition of a pseudocount and correction of batch effects was performed using the ComBat function from the sva R-package [20].

### 2.5. Statistical Analyses

A multivariable conditional logistic regression model was employed to estimate the risk of PCa for each metabolite. The analyses were conditioned on the matching variables and further adjusted for smoking status (never, past, current, unknown), physical activity (inactive, moderately active, active, unknown), educational level (none, primary, technical/professional, secondary, longer, unknown), marital status (married or cohabiting, single or not cohabiting), body mass index (BMI) (<22.5, 22.5–24.9, 25–29.9, ≥30.0 kg/m^2^), and alcohol intake (non-consumers, <5, 5.0–14.9, 15–29.9, ≥30.0 g/day). Resulting *p* values were corrected with the Benjamini–Hochberg false discovery rate (FDR) method to account for multiple testing. Spearman’s correlation coefficient was used to assess the correlation between metabolite concentrations in plasma and the habitual baseline intake (g/d) of 30 selected foods and food groups (Supplementary Table S3). The statistical software of choice for all analyses was R (R software, Vienna, Austria) version 4.2.2.

## 3. Results

### 3.1. Characteristics of Participants

The current study included 851 pairs of first incident PCa cases and matched controls (1:1). The mean age (SD) of participants at blood collection was 55.4 (7.06) years, and the mean time from blood collection to PCa diagnosis among cases was 14 years. There were no marked differences in baseline characteristics between the cases and controls (Table 1). According to the stage and grade of the disease, PCa cases were mostly localized (n = 303) and low–intermediate grade (n = 589), representing 35.6% and 69.2% of the total number of PCa cases, respectively (Table 1). A total of 57 PCa cases (6.70%) resulted in a fatal PCa outcome. The distribution of metabolite concentrations by case–control status is shown in Supplementary Table S1.

### 3.2. Associations Between Plasma Metabolites and PCa Risk

A total of 31 metabolites of the 146 included for multivariate conditional logistic regression models were associated with the risk of PCa (*p* ≤ 0.05), but none of the associations were statistically significant after controlling for multiple testing (Supplementary Table S2). Specifically, 15 and 16 metabolites were inversely and positively associated with the risk of PCa overall and by clinical subtypes, respectively. Metabolites of note were predominately of exogenous origin, including derivatives of (poly)phenols (16), amino acids (9), amines (1), organic acids (1), organosulfur compounds (1), food additives (1), xanthine alkaloids (1), and furan compounds (1).

#### 3.2.1. Metabolites and Risk of Overall PCa Risk

**Figure 1:**
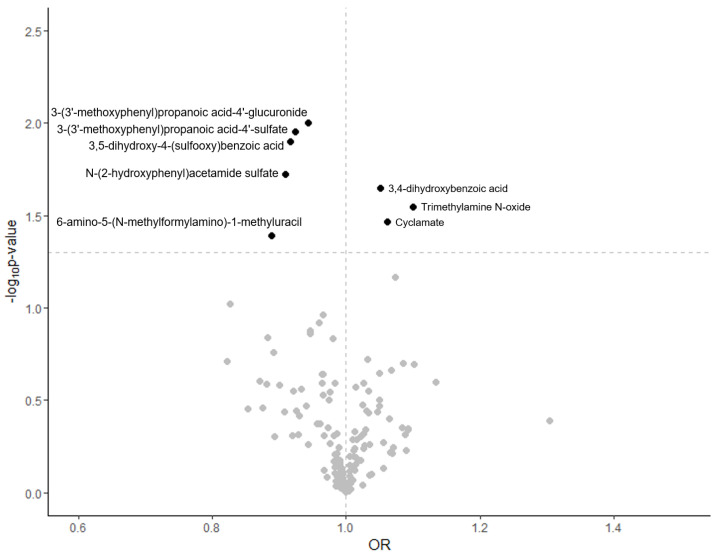
Volcano plot showing the magnitude of associations (odds ratio [OR]) and statistical significance (−log10 *p* values) for plasma metabolites and overall prostate cancer risk in the nested case–control within the EPIC cohort. Significant associations (above the dotted horizontal line) were set at −log10 *p*-values ≥ −1.30 (*p* ≤ 0.05).

**Figure 2:**
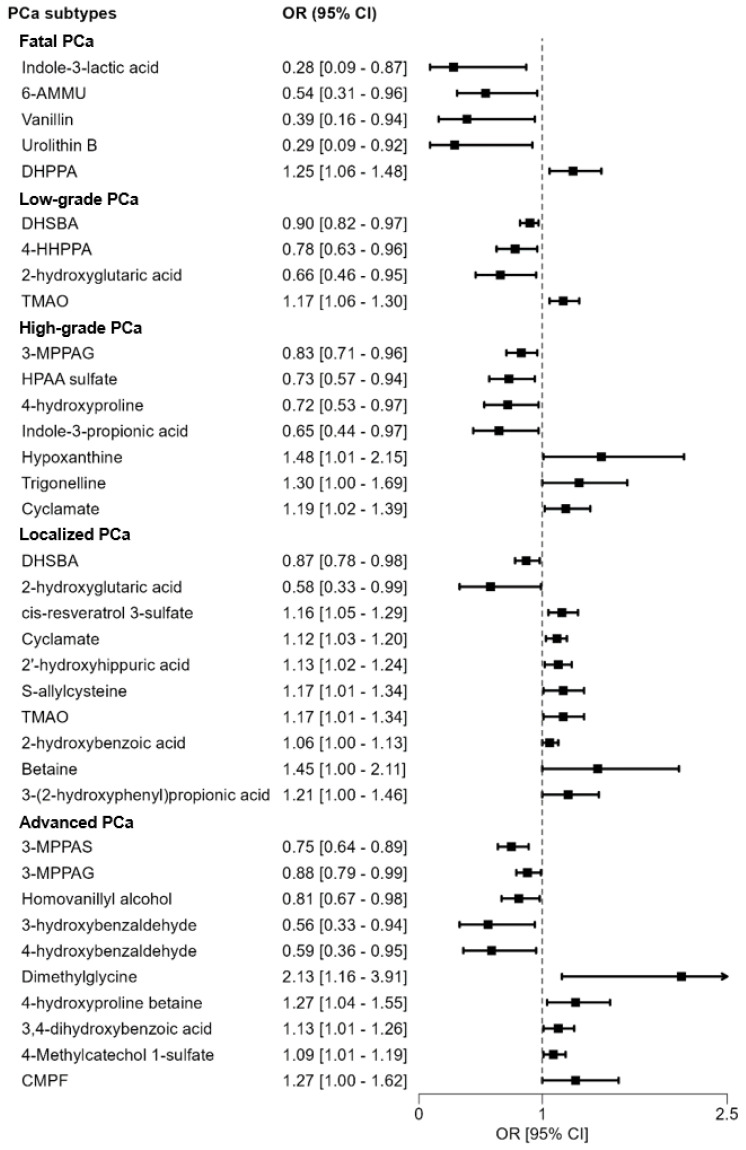
Volcano forest plot showing odds ratios and 95% CI for the associations between plasma metabolites and five relevant clinical tumor subtypes of prostate cancer in the nested case–control study within the EPIC cohort. Only significant associations at *p* < 0.05 (not FDR adjusted) are shown. Abbreviations: 3-MPPAG = 3-(3′-methoxyphenyl)propanoic acid-4′-glucuronide; 3-MPPAS = 3-(3′-methoxyphenyl)propanoic acid-4′-sulfate; 4-HHPPA = (R/S)-2-hydroxy-3-(4′-hydroxyphenyl)propanoic acid; 6-AMMU = 6-amino-5-(N-methylformylamino)-1-methyluracil; CMPF = 3-carboxy-4-methyl-5-propyl-2-furanpropionic acid; DHPPA = 3-(3′,4′-dihydroxyphenyl)propanoic acid; DHSBA = 3,5-dihydroxy-4-(sulfooxy)benzoic acid; HPAA sulfate = N-(2-hydroxyphenyl)-acetamide sulfate; TMAO = trimethylamine N-oxide.

Significant associations (*p* ≤ 0.05) between plasma metabolites and the risk of overall PCa are shown in Figure 1. Men with higher concentrations of cyclamate, trimethylamine N-oxide (TMAO), and 3,4-dihydroxybenzoic acid had a higher risk of overall PCa, while lower risk of PCa was seen with higher concentrations of 3,5-dihydroxy-4-(sulfooxy)benzoic acid, 3-(3′-methoxyphenyl)propanoic acid-4′-glucuronide, 6-amino-5-(N-methylformylamino)-1-methyluracil (6-AMMU), N-(2-hydroxyphenyl)acetamide sulfate (HPAA sulfate), and 3-(3′-methoxyphenyl)propanoic acid-4′-sulfate. The strongest positive and strongest negative associations were for TMAO and 6-AMMU with overall PCa risk (OR = 1.10; 95% CI 1.01–1.20; and OR = 0.89; 95% CI 0.79–1.00, respectively). (Supplementary Table S2 and Figure 2).

All metabolites associated with overall PCa risk overlapped with the risk of at least one PCa subtype in the same direction (Supplementary Table S2 and Figure 2).

#### 3.2.2. Metabolites and Risk of Clinical Subtypes of PCa

The metabolites positively and inversely associated with the risk of each PCa clinical subtype at a *p* < 0.05 are shown in Figure 2. According to the odds ratios and 95% CI, the strongest positive and inverse associations (respectively) were seen for higher plasma concentrations of 3-(3′,4′-dihydroxyphenyl)propanoic acid (OR = 1.25; 95% CI 1.06–1.48) and indole-3-lactic acid (OR = 0.28; 95% CI 0.09–0.87) with fatal PCa; TMAO (OR = 1.17; 95% CI 1.06–1.30) and 2-hydroxyglutaric acid (OR = 0.66; 95% CI 0.46–0.95) with low-grade PCa; hypoxanthine (OR = 1.48; 95% CI 1.01–2.15) and indole-3-propionic acid (OR = 0.65; 95% CI 0.44–0.97) with high-grade PCa; betaine (OR = 1.45; 95% CI 1.00–2.11) and 2-hydroxyglutaric acid (OR = 0.58; 95% CI 0.33–0.99) with localized PCa; and dimethylglycine (OR = 2.13; 95% CI 1.16–3.91) and 3-hydroxybenzaldehyde (OR = 0.56; 95% CI 0.33–0.94) with advanced PCa.

### 3.3. Correlations Between Metabolites and Dietary Intakes

**Figure 3:**
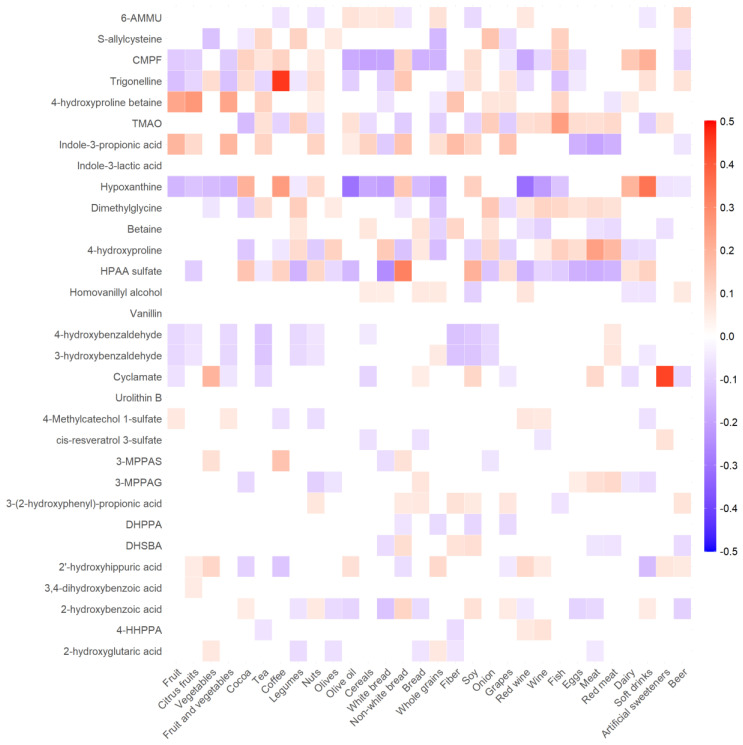
Heatmap showing significant (*p* < 0.05) Spearman’s correlations between plasma concentrations of 31 metabolites and the habitual intake of selected foods and food groups in the nested prostate cancer case–control study within the EPIC cohort. Full data on rho coefficients and statistical significance for each pair of correlations are shown in Supplementary Table S2. Abbreviations: 3-MPPAG = 3-(3′-methoxyphenyl)propanoic acid-4′-glucuronide; 3-MPPAS = 3-(3′-methoxyphenyl)propanoic acid-4′-sulfate; 4-HHPPA = (R/S)-2-hydroxy-3-(4′-hydroxyphenyl)propanoic acid; 6-AMMU = 6-amino-5-(N-methylformylamino)-1-methyluracil; CMPF = 3-carboxy-4-methyl-5-propyl-2-furanpropionic acid; DHPPA = 3-(3′,4′-dihydroxyphenyl)propanoic acid; DHSBA = 3,5-dihydroxy-4-(sulfooxy)benzoic acid; HPAA sulfate = N-(2-hydroxyphenyl)-acetamide sulfate; TMAO = trimethylamine N-oxide.

Spearman’s correlations between plasma metabolites and the usual intake of selected foods and food groups are shown in Figure 3 and Supplementary Table S3. All individual metabolites were not correlated or weakly correlated with the individual and combined intake of foods, showing in the case of positive correlations rho coefficients between 0.05 (*p* = 0.05) and 0.46 (*p* < 0.001). The strongest correlations were for trigonelline and hypoxanthine ((rho = 0.46) with coffee (rho = 0.25), cyclamate with artificial sweeteners (rho = 0.44), HPAA sulfate with non-white bread (rho = 0.32), 4-hydroxyproline betaine with citrus fruits (rho = 0.27), 4-hydroxyproline with meat (rho = 0.25), and TMAO (rho = 0.25) and 3-carboxy-4-methyl-5-propyl-2-furanpropionic acid (CMPF) (rho = 0.20) with fish. All the above-described correlations had a *p* value < 0.001.

## 4. Discussion

In the current study, we used a comprehensive targeted metabolomics approach to assess the association of plasma concentrations of almost 150 metabolites with PCa risk in 851 matched PCa case–control pairs of participants of the EPIC cohort. Although associations between 31 metabolites and risk of PCa, including overall and relevant clinical subtypes, were found, these did not survive correction for multiple testing. The metabolites included endogenous and diet-related compounds from several metabolite classes. Additionally, some metabolites were derived from microbial metabolism, highlighting the potential role of gut microbiota in PCa carcinogenesis. Our findings, if validated in future studies, could be used to improve the detection of men at increased risk of PCa through the development of novel risk calculators [21] or nomograms [22].

Several previous studies using metabolomics to assess the prospective associations between circulating metabolites and PCa risk have been published. The majority of these, however, have focused on the analysis of metabolites of endogenous origin [8,9,10,23,24,25,26,27] and/or the use of overall risk of PCa as the main outcome [24,25,27]. This meant that the analysis of exogenous metabolites and other PCa clinical subtypes, including mortality, have been largely unexplored. In the framework of the EPIC cohort, targeted metabolomic studies have previously investigated the associations between prediagnostic levels of circulating endogenous metabolites and the overall risk of PCa, by tumor characteristics, and mortality [8,9,10,11,23]. To the best of our knowledge, none of those studies have analyzed a large panel of exogenous metabolites and elucidated their association with PCa risk.

### 4.1. Endogenous Metabolites and PCa Risk

Endogenous metabolites associated with PCa risk identified in our study included derivatives of amino acid metabolism, nucleotide metabolism, organic acids, and amines.

Among amino acid derivatives, we identified negative associations for indole-3-lactic acid and indole-3-propionic acid with risk of fatal and high-grade PCa, respectively, although these associations were no longer significant after correction for multiple testing. These results contrast with those reported by Mondul et al. [28], who despite finding inverse associations for two indole metabolites, indole-3-propionic acid and indole-3-butyric acid, they were with regard to the risk of a nonaggressive PCa subtype. We observed a suggestion of negative associations for (R/S)-2-hydroxy-3-(4′-hydroxyphenyl)propanoic acid and 4-hydroxyproline with low-grade and high-grade PCa risk, respectively. To our knowledge, no previous study has reported the negative association between (R/S)-2-hydroxy-3-(4′-hydroxyphenyl)propanoic acid and PCa risk. On the other hand, an inverse association between a closely related metabolite, 4-hydroxyphenylpyruvic acid, and risk of lethal PCa was previously reported in the ATBC (Alpha-Tocopherol, Beta-Carotene Cancer Prevention) study by Huang et al. [29].

The result for 4-hydroxyproline is partially opposite to the result published by Schmidt et al. within the EPIC cohort [9]. These authors reported that plasma concentrations of trans-4-hydroxyproline were inversely associated with overall PCa risk; however, this association was not statistically significant after controlling for multiple testing. High concentrations of betaine, dimethylglycine, and TMAO were positively associated with advanced and localized PCa risk in our study, although associations did not remain after FDR correction. These results contrast with the null associations reported between these three metabolites and the overall and lethal PCa risk in nested case–control studies within the JANUS [27] and PLCO (Prostate, Lung, Colorectal, and Ovarian Cancer Screening) [30] cohorts, respectively. However, they are in agreement with the positive association between serum TMAO and aggressive PCa risk observed in a nested case–control study in the ATBC study [28]. Betaine, dimethylglycine, and TMAO are systemic breakdown products of dietary choline, whose serum concentrations have also been associated with an increased risk of lethal PCa [30]. Our results, therefore, appear to confirm previous research suggesting that the choline metabolism pathway may modulate PCa risk, especially the lethal outcome [31]. In the present study, we found 2-hydroxyglutaric acid to have a negative association with future PCa risk for both low-grade and localized PCa subtypes. This association is opposite to that found by Huang et al. [32], who observed a positive association between 2-hydroxyglutaric acid and PCa-specific mortality among those in the ATBC study. The positive association between hypoxanthine and high-grade PCa risk in the present study contrasts with the negative associations found for this metabolite and the purine derivatives N1-methylinosine, AMP, and ADP with the risk of fatal PCa in a case–cohort study of the Cancer Prevention Study-II Nutrition Cohort [33]. Our finding would confirm that dysregulation in purine metabolism may precede the onset of PCa.

### 4.2. Diet- and Microbial-Related Metabolites and PCa Risk

To the best of our knowledge, this is the first large prospective study to assess the association between exogenous metabolites and PCa risk. Metabolites comprising the diet-related panel associated with PCa risk in the current study were predominantly derivatives of plant-based foods, especially (poly)phenol by-products. To our knowledge, epidemiological evidence on prospective associations between (poly)phenols and PCa risk is scarce and is limited to the use of dietary exposure biomarkers [34,35,36] and systemic concentrations of isoflavones and lignans [37]. Overall, the results from observational studies using dietary (poly)phenols exposure reported null or inconclusive associations with PCa risk [34,35,36]. Regarding isoflavones and lignans, although we included several compounds from these (poly)phenol subclasses for their analysis, none of them were found to be significantly associated with PCa risk. These null results, therefore, should be confirmed in further studies using nutritional biomarkers.

In accordance with the previous literature, we found some metabolites weakly to moderately correlated with the habitual intake of potential food sources (Supplementary Table S3). Putative biomarkers of plant-based foods, such as HPAA sulfate for whole grains [38] and 4-hydroxyproline betaine for citrus fruit [39], were associated with a reduced risk of overall and high-grade PCa, respectively, while trigonelline for coffee [40] consumption had an increased risk of high-grade PCa. These associations are intriguing, especially for citrus fruit [41] and whole grains [42], whose intake have been reported to have no association with PCa risk. Among putative biomarkers of non-plant-based foods, we observed positive associations for cyclamate and TMAO with the risk of overall, low-grade, high-grade, and localized PCa risks. Some artificial sweeteners, especially aspartame and acesulfame-K, are suggested to be related to a higher cancer risk [43], but not cyclamate [44]. To the best of our knowledge, this is the first study suggesting that an increased concentration of plasma cyclamate may be associated with a higher risk of PCa. This finding, therefore, warrants further research. On the other hand, circulating TMAO was consistently associated with animal protein intake in a pooled analysis of 16 population-based studies [45]. To date, there is still not enough evidence supporting a clear positive association between animal protein intake and PCa incidence at the epidemiological level [46].

The observed associations between several microbial metabolites and PCa risk in the current study suggest that host microbiota might play an important role in prostate carcinogenesis and tumor progression [47]. For example, indole-3-lactic acid, which is produced by the microbial metabolism of tryptophan, showed the strongest inverse association with PCa risk, especially a fatal outcome. Observational and intervention studies in humans have shown that intestinal microbiota may play a crucial role in the occurrence and progression of PCa through short-chain fatty acids, testosterone, estrogen, folic acid, and phenylacetylglutamine metabolites [48]. Although we did not include any of these metabolites, our study is the first to include a considerable number of microbial metabolites from endogenous and dietary origin and to report the prospective associations between these metabolites and several PCa clinical subtypes. Noteworthy, the observed associations for the microbial metabolites of (poly)phenols highlight the probiotic role of these phytochemicals in relation to PCa risk. Increasing evidence suggests that altering the gut microbiome using prebiotic or probiotic interventions may prevent or delay prostate cancer development [49].

The major strength of our study is, on the one hand, the use of a validated high-throughput and comprehensive targeted metabolomics method [15]. We were able to analyze in a robust way a large set of almost 600 metabolites of different origins in a large number of samples (891 matched pairs). Additionally, the large sample size of the prospective study design, and the coverage of several European countries, makes this study somewhat more complete and comprehensive compared to metabolomics studies that used smaller sample sizes and lower country heterogeneity [24,26,32]. Our study also has limitations. Firstly, none of our findings reached the multiple testing correction (FDR) threshold, so they need to be interpreted with caution. Secondly, we relied on data collected from self-reported dietary questionnaires for food intake information. In this sense, although dietary questionnaires were validated in each center/country [50], their use may have led to measurement errors, especially in food intake estimation, which may affect the tested correlations between food intake and plasma metabolites. In view of these limitations, our findings therefore should be interpreted with caution, especially those associations between metabolites and PCa outcomes that are reported for the first time.

## 5. Conclusions

In conclusion, the results from the current nutrimetabolomics study suggest that the plasma concentrations of several endogenous, dietary, and microbial metabolites might prospectively be related to PCa risk. Noteworthy, our study generates new hypotheses to investigate the role of diet and gut microbiota in PCa. In order to provide greater high-quality epidemiological evidence on PCa risk, our results need to be further replicated in other large prospective studies.

## Figures and Tables

**Table 1:** Baseline characteristics of participants.

Characteristics	All (N = 1702)	Cases (n = 851)	Controls (n = 851)
Age at blood collection, years (SD)	55.4 (7.06)	55.3 (7.06)	55.4 (7.06)
Body mass index, kg/m^2^ (SD) ^1^	26.7 (3.43)	26.6 (3.24)	26.9 (3.60)
Body mass index, kg/m^2^ n (%) ^1^			
<22.5	130 (7.60)	67 (7.90)	63 (7.40)
22.5–24.9	339 (19.9)	172 (20.2)	167 (19.6)
25–29.9	927 (54.5)	473 (55.6)	454 (53.3)
≥30.0	306 (18.0)	139 (16.3)	167 (19.6)
Smoking status, n (%) ^1^			
Never	559 (32.8)	297 (34.9)	262 (30.8)
Former	665 (39.1)	323 (38.0)	342 (40.2)
Current	458 (26.9)	217 (25.5)	241 (28.3)
Alcohol intake, n (%) ^1^			
Non-consumers	164 (9.60)	83 (9.80)	81 (9.5)
<5.0 g/day	317 (18.6)	156 (18.3)	161 (18.9)
5.0–14.9 g/day	371 (21.8)	187 (22.0)	184 (21.6)
15.0–29.9 g/day	358 (21.0)	186 (21.9)	172 (20.2)
≥30.0 g/day	492 (28.9)	239 (28.1)	253 (29.7)
Physical activity, n (%) ^1^			
Inactive	355 (20.9)	179 (21.0)	176 (20.7)
Moderately inactive	535 (31.4)	263 (30.9)	272 (32.0)
Moderately active	405 (23.8)	199 (23.4)	206 (24.2)
Active	369 (21.7)	193 (22.7)	176 (20.7)
Marital status, n (%) ^1^			
Married or cohabiting	1569 (92.2)	787 (92.5)	782 (91.9)
Not married or cohabiting	133 (7.80)	64 (7.50)	69 (8.10)
Educational level, n (%) ^1^			
None	181 (10.6)	95 (11.2)	86 (10.1)
Primary or equivalent	545 (32.0)	268 (31.5)	277 (32.6)
Secondary	230 (13.5)	113 (13.3)	117 (13.7)
Technical/Professional	338 (19.9)	167 (19.6)	171 (20.1)
Longer (University)	327 (19.2)	164 (19.3)	163 (19.2)
Cases only			
Age at diagnosis, years (SD) ^1^		69.2 (7.0)	
Time to diagnosis, years (SD) ^1,2^		14.0 (2.18)	
Stage, n (%)			
Localized		303 (35.6)	
Advanced		157 (18.4)	
Grade			
Low–intermediate grade		589 (69.2)	
High grade		116 (13.6)	
Death from prostate cancer, n (%)		57 (6.70)	

^1^ Unknown values for some participants; the calculations of percentages exclude missing values. ^2^ Time between blood collection and diagnosis.

## Data Availability

For information on how to submit an application for gaining access to EPIC data and/or biospecimens, please follow the instructions at http://epic.iarc.fr/access/ (accessed on 15 October 2024).

## Supplementary Materials

The following supporting information can be downloaded at: https://www.mdpi.com/article/10.3390/cancers16234116/s1, Figure S1: flowchart showing the number of metabolites available in each preprocessing step; Table S1: distribution of metabolite concentrations by PCa cases and controls. Table S2: odds ratios and 95% CI for overall and six relevant clinical subtypes of prostate cancer risk by log2 concentration of 147 plasma metabolites in a nested case–control study within the EPIC cohort; Table S3: correlation coefficients for the relationship between plasma concentrations of 31 metabolites and the habitual intake of selected foods and food groups in a nested prostate cancer case–control study.
