# Supplementary material for: Prediagnostic Plasma Nutrimetabolomics and Prostate Cancer Risk: A Nested Case–Control Analysis Within the EPIC Study
Source: Cancers (Basel). 2024 Dec 8;16(23):4116. doi: 10.3390/cancers16234116 (PMC11639937; doi:10.3390/cancers16234116)
Supplement: Supplementary file 1 [file cancers-16-04116-s001.zip › Supplementary Figure S1.pdf]

Supplementary Figure S1. Flowchart showing the number of metabolites available in each pre-processing step.

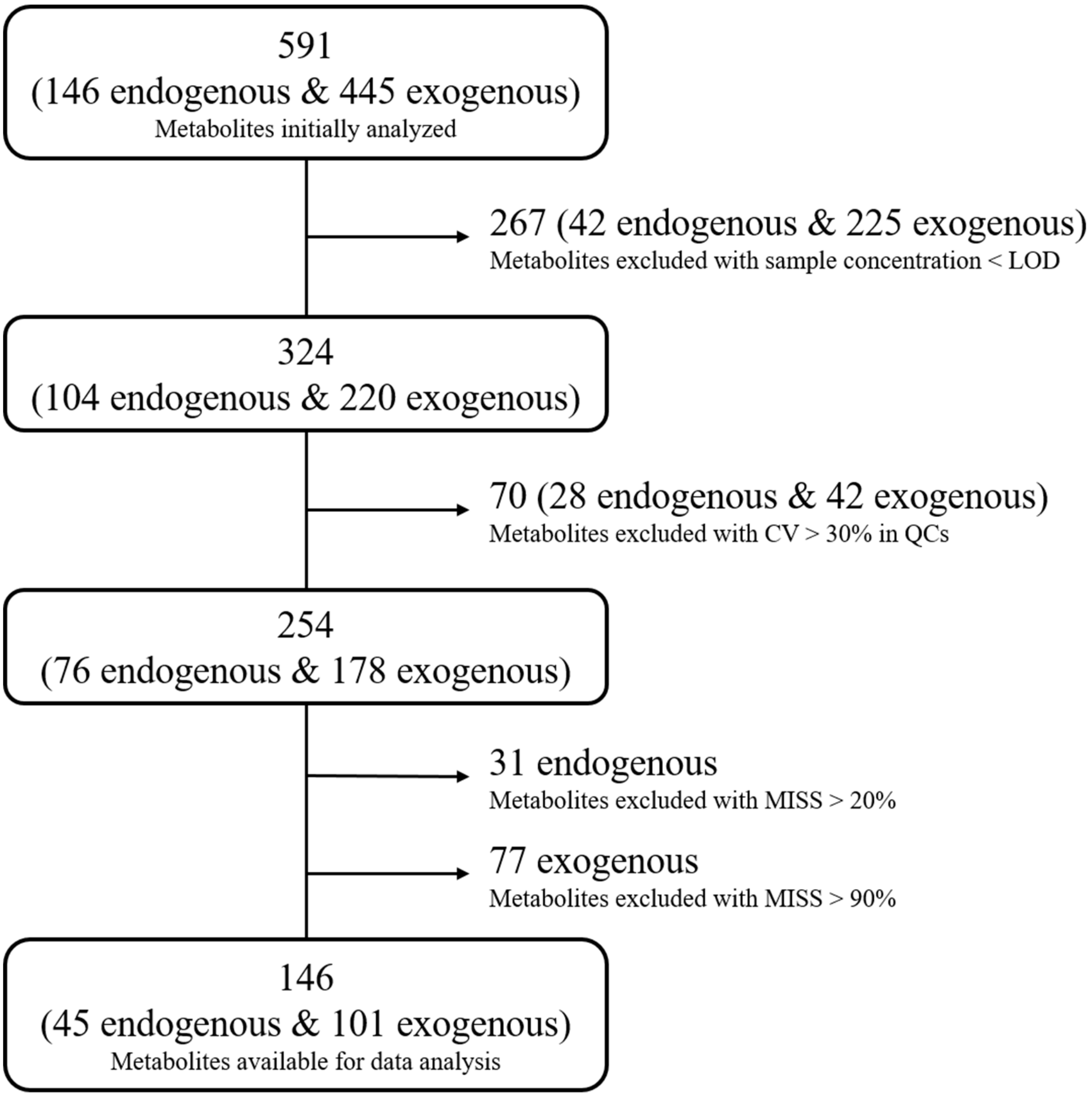

Abbreviations: CV, coefficient of variation; LOD, limit of detection used for chromatography analysis; MISS, missing values; QCs, quality control samples.
